# Effects of the PPAR-β/δ agonist GW0742 during resuscitated porcine septic shock

**DOI:** 10.1186/2197-425X-1-9

**Published:** 2013-10-29

**Authors:** Martin Wepler, Sebastian Hafner, Angelika Scheuerle, Matthias Reize, Michael Gröger, Florian Wagner, Florian Simon, José Matallo, Frank Gottschalch, Andrea Seifritz, Bettina Stahl, Martin Matejovic, Amar Kapoor, Peter Möller, Enrico Calzia, Michael Georgieff, Ulrich Wachter, Josef A Vogt, Christoph Thiemermann, Peter Radermacher, Oscar McCook

**Affiliations:** Sektion Anästhesiologische Pathophysiologie und Verfahrensentwicklung, Klinik für Anästhesiologie, Universitätsklinikum, Helmholtzstrasse 8-1, Ulm, 89081 Germany; Abteilung Pathologie, Universitätsklinikum, Ulm, 89081 Germany; Klinik für Gefäß- und Endovaskularchirurgie, Universitätsklinikum, Heinrich-Heine-Universität, Düsseldorf, 40225 Germany; 1. Interni klinika, Lekarska fakulta a Fakultni nemocnice, Karlova univerzita Praha, Plzeň, 304 60 Czech Republic; Barts and The London School of Medicine and Dentistry, William Harvey Research Institute, Centre for Translational Medicine and Therapeutics, Queen Mary University of London, London, EC1M 6BQ UK

**Keywords:** Kidney function, Neutrophil gelatinase-associated lipocalin, Apoptosis, Necrosis, Heme oxygenase-1, Oxidative stress, Inducible nitric oxide synthase, Nitrotyrosine, Nuclear transcription factor κB

## Abstract

**Background:**

In un-resuscitated rodent models of septic shock, the peroxisome proliferator-activated receptor-β/δ (PPAR-β/δ) agonist GW0742 improved visceral organ function. Therefore, we tested the hypothesis whether GW0742 would attenuate kidney injury during long-term, resuscitated, porcine polymicrobial septic shock.

**Methods:**

Six, 12, and 18 h after the induction of fecal peritonitis by inoculation of autologous feces, anesthetized, mechanically ventilated, and instrumented male pigs with pre-existing atherosclerosis resulting from familial hypercholesteremia and atherogenic diet randomly received either vehicle (dimethyl sulfoxide, *n* = 12) or GW0742 (*n* = 10). Resuscitation comprised hydroxyethyl starch and norepinephrine infusion titrated to maintain mean arterial pressure at baseline values.

**Results:**

Despite aggressive fluid resuscitation, fecal peritonitis was associated with arterial hypotension requiring norepinephrine infusion, ultimately resulting in progressive lactic acidosis and acute kidney injury. GW0742 did not beneficially affect any parameter of systemic and regional hemodynamics, gas exchange, metabolism, or organ function. The parameters of inflammation, oxidative and nitrosative stress, and organ injury (post-mortem analysis for histomorphology and markers of apoptosis) were not influenced either. Immunohistochemistry of pre-shock kidney biopsies from a previous study in this swine strain showed markedly lower PPAR-β/δ receptor expression than in healthy animals.

**Conclusions:**

In swine with pre-existing atherosclerosis, the PPAR-β/δ agonist GW0742 failed to attenuate septic shock-induced circulatory failure and kidney dysfunction, most likely due to reduced receptor expression coinciding with cardiovascular and metabolic co-morbidity.

**Electronic supplementary material:**

The online version of this article (doi:10.1186/2197-425X-1-9) contains supplementary material, which is available to authorized users.

## Background

Ample evidence is available that the activation of the peroxisome proliferator-activated receptors (PPAR), ligand-activated transcription factors of the nuclear hormone receptor family, presenting as PPAR-α, PPAR-γ, and PPAR-β/δ, has beneficial effects in various shock models. The highly selective synthetic PPAR-β/δ agonist GW0742 [1] blunted shock-induced organ injury as a result of attenuated inflammation and oxidative and nitrosative stress and decreased activation of the nuclear transcription factor κB (NF-κB) [2–8]. These organ-protective properties were also present in animals with obesity [9] and diabetes [10], most likely as a result of enhanced insulin sensitivity and, consequently, improved glucose utilization [11], as well as attenuated endothelial dysfunction [12]. However, all these data originate from short-term, un-resuscitated rodent models characterized by hypotension and low cardiac output. Therefore, we tested the hypothesis whether GW0742 may attenuate kidney dysfunction during long-term, resuscitated, porcine fecal peritonitis-induced septic shock [13, 14]. Given the beneficial effects of GW0742 on glucose homoeostasis [11] and vascular function [12], we investigated swine with hyperlipidemia and ubiquitous atherosclerosis [15].

## Methods

The University of Ulm Animal Care Committee and the Federal authorities for animal research had approved the experiments, which were performed in adherence to National Institutes of Health Guidelines on the Use of Laboratory Animals. Twenty-two adult, castrated, male pigs (age 15 to 30 months, median (interquartile range) body weight of 72 (65 to 81) kg) were used. The pig strain is a cross-breed of Rapacz farm pigs homozygous for the R84C low-density lipoprotein (LDL) receptor mutation with smaller strains ('FBM’), with hypercholesteremia due to an atherogenic diet [15].

### Animal preparation

Anesthesia and surgical instrumentation have been described in detail previously [13–15]. Briefly, anesthesia was induced with atropine, propofol, and ketamine to allow endotracheal intubation and was maintained thereafter with pentobarbitone, buprenorphine, and pancuronium. Ventilator settings were fraction of inspired O_2_ (FiO_2_) 0.35, positive end-expiratory pressure (PEEP) 10 cmH_2_O, tidal volume 8 mL·kg^-1^, respiratory rate 10 to 12 breaths·min^-1^ adjusted to maintain arterial PCO_2_ = 35 to 40 mmHg, inspiratory (*I*)/expiratory (*E*) ratio 1:1.5, peak airway pressure <40 cmH_2_O, and modified to *I*/*E* ratio 1:1 and PEEP 12 or 15 cmH_2_O, respectively, if the ratio of arterial O_2_ partial pressure (PaO_2_)/FiO_2_ is <300 or <200 mmHg [13–15]. The right jugular vein and carotid artery were exposed for the insertion of a central venous catheter sheath and the placement of a balloon-tipped pulmonary artery catheter to measure central venous (CVP), pulmonary arterial (MPAP), and pulmonary artery occlusion pressures (PAOP), and a thermistor-tipped arterial catheter for blood pressure (MAP) recording and transpulmonary single indicator thermodilution-cardiac output (CO) measurement. The right kidney and a femoral vein were surgically exposed, and a catheter was advanced into the inferior vena cava and manually guided into a right renal vein under visual control [14, 15]. A catheter in the urinary bladder allowed urine collection. Two tubes were placed through the abdominal wall for peritonitis induction. Ringer's solution was continuously infused as maintenance fluid (10 mL·kg^-1^·h^-1^). As needed, animals received hydroxyethyl starch to maintain cardiac filling pressures during surgery.

### Experimental protocol

After instrumentation and an 8-hour recovery, baseline data were collected. Thereafter, the supernatant (3 mL·kg^-1^) of 1.0 g·kg^-1^ autologous feces incubated in 500 mL 0.9% saline for 12 h at 38°C was injected into the abdominal cavity via the abdominal drainage tubes. Hydroxyethyl starch (10 mL∙kg^-1^∙h^-1^, 5 mL∙kg^-1^∙h^-1^ if CVP or PAOP is >18 mmHg) allowed maintaining hyperdynamic hemodynamics. If necessary, norepinephrine was infused and titrated to maintain MAP at baseline values (no further increase if the heart rate is ≥160 min^-1^ to avoid tachycardia-induced myocardial ischemia) [13–16]. Animals randomly received i.v. GW0742 (0.03 mg∙kg^-1^, *n* = 10, body weight 71 (66 to 80) kg) or vehicle (DMSO; *n* = 12, body weight 75 (66 to 81) kg) at 6, 12, and 18 h after the induction of peritonitis. The same GW0742 dose attenuated renal dysfunction in murine endotoxic shock [2] and organ injury after kidney ischemia/reperfusion injury in diabetic rats [10]. The timing of the GW0742 administration was chosen, because10-day survival was doubled in mice injected with this dose at 6.5 and 12.5 h after cecal ligation and puncture-induced sepsis [2]. After additional data collection at 12 and 24 h after the induction of peritonitis, animals were sacrificed under deep anesthesia.

### Measurements and calculations

Hemodynamics, gas exchange (calorimetric O_2_ uptake and CO_2_ production, arterial and mixed venous blood gases), glucose, lactate, creatinine, renal venous nitrite + nitrate (NO_2_^-^ + NO_3_^-^), tumor necrosis factor-α (TNFα), and interleukin-6 (IL-6) concentrations were determined as described previously [13–16]. Endogenous glucose production and glucose oxidation were derived from plasma 1,2,3,4,5,6-^13^C_6_-glucose and the mixed expiratory ^13^CO_2_ isotope enrichment, respectively, during continuous glucose isotope infusion [13, 14]. Urinary and blood creatinine and Na^+^ levels were analyzed to calculate creatinine clearance and fractional Na^+^-excretion [9] together with blood neutrophil gelatinase-associated lipocalin (NGAL) [15]. At the end of the experiment, immediate *post-mortem* kidney tissue samples were analyzed for the expression of the inducible nitric oxide synthase (iNOS), heme oxygenase-1 (HO-1), and cleaved caspase-3 as well as for the activation of the nuclear transcription factor κB (NF-κB) as described in detail previously [15, 16]. Pyramid-shaped kidney specimens showing kidney cortex, medulla, renal papilla, and the corresponding renal calyx were dissected for histopathological examination, performed by an experienced pathologist (A.S.) blinded for the sample grouping [15]. Histopathological alterations were analyzed for the degree of 'glomerular tubularization’, dilatation of Bowman's space, and swelling of Bowman's capsule, cellular edema of the proximal tubule, distal tubular dilatation and elongation, tubular protein cylinders, and tubular necrosis as described in detail previously [15].

Immunohistochemistry allowed quantifying the formation of nitrotyrosine (rabbit anti-Nitrotyrosine, Millipore, Schwalbach, Germany) [16] and the expression of the PPAR-β/δ (rabbit anti-PPAR delta antibody #ab23673, Abcam plc, Cambridge, UK), the method being described in detail in the supplement. The latter was determined on formalin-fixed, paraffin-embedded kidney biopsies, which had been taken during surgical instrumentation both in FBM and young and healthy German Landswine in a previous study [15], as well as age-matched FBM pigs that had not been fed with the atherogenic diet (*n* = 5 in each group). Results are presented as mean densitometric sum red.

### Statistical analysis

Data are presented as median (quartiles). After the exclusion of normal distribution using the Kolmogorov-Smirnoff test, differences within groups were analyzed by a Friedmann analysis of variance on ranks and a subsequent Dunn's test with Bonferroni correction. Inter-group differences were tested using a Mann–Whitney rank sum test.

## Results

One animal in the control group died 15 h after the induction of peritonitis; therefore, data at the end of the experiment originate from 11 vehicle-treated animals only. Colloid and norepinephrine requirements were comparable in the two groups (Additional file 1: Table A). MAP progressively decreased and CO increased despite aggressive circulatory support, ultimately resulting in impaired pulmonary gas exchange and lactic acidosis, however, without inter-group difference (Table 1). Sepsis caused a progressive deterioration of renal function, coinciding with increased renal venous concentrations of pro-inflammatory cytokines, NO metabolites, and isoprostanes, again without any inter-group difference (Table 2). Western blotting confirmed the findings on blood biomarkers, while tissue expression of HO-1, iNOS, and activated caspase 3 were comparable to those from animals that had undergone surgical instrumentation only, sepsis nearly tripled NF-κB activation (Additional file 1: Table B). There was, however, no treatment effect.

**Table 1:** **Systemic hemodynamics, gas exchange, and metabolism**

		Before peritonitis	12-h peritonitis	24-h peritonitis
Body temperature (°C)	DMSO	36.5 (35.7, 37.4)	37.6 (37.0, 38.4)^a^	38.4 (37.5, 38.7)^a^
	GW0742	36.9 (36.5, 37.3)	38.1 (37.7, 38.2)^a^	38.0 (37.7, 38.3)^a^
Heart rate (min^-1^)	DMSO	83 (70, 99)	144 (121, 149)^a^	149 (142, 163)^a^
	GW0742	69 (63, 92)	134 (126, 146)^a^	158 (150, 162)^a^
Mean arterial pressure (mmHg)	DMSO	103 (92, 108)	90 (87, 101)^a^	75 (65, 98)^a^
	GW0742	110 (106, 114)	97 (93, 100)^a^	83 (75, 94)^a^
Mean pulmonary artery pressure (mmHg)	DMSO	25 (22, 26)	38 (28, 42)^a^	39 (35, 40)^a^
	GW0742	27 (24, 27)	35 (34, 38)^a^	42 (38, 47)^a^
Central venous pressure (mmHg)	DMSO	13 (9, 14)	15 (12, 17)^a^	18 (17, 18)^a^
	GW0742	13 (11, 15)	15 (13, 17)^a^	19 (17, 22)^a^
Pulmonary artery occluded pressure (mmHg)	DMSO	13 (10, 14)	15 (14, 17)^a^	17 (17, 19)^a^
	GW0742	15 (11, 17)	16 (14, 18)^a^	17 (16, 20)^a^
Cardiac output (L·min^-1^)	DMSO	4.4 (3.8, 5.2)	6.4 (5.7, 7.9)^a^	8.0 (5.7, 9.3)^a^
	GW0742	4.4 (3.7, 5.2)	7.1 (6.1, 8.5)^a^	7.6 (7.1, 7.8)^a^
Hemoglobin (g·L^-1^)	DMSO	94 (80, 100)	111 (99, 123)^a^	115 (110, 121)^a^
	GW0742	91 (80, 102)	104 (107, 119)^a^	110 (107, 119)^a^
Arterial PO_2_ (mmHg)	DMSO	176 (161, 185)	151 (121, 161)	118 (91, 144)^a^
	GW0742	180 (171, 181)	163 (125, 172)	118 (110, 139)^a^
PaO_2_/FiO_2_ ratio (mmHg)	DMSO	550 (502, 583)	459 (362, 503)^a^	244 (109, 398)^a^
	GW0742	536 (487, 611)	470 (392, 574)^a^	236 (205, 386)^a^
Arterial PCO_2_ (mmHg)	DMSO	36 (34, 38)	38 (37, 41)	37 (35, 40)
	GW0742	35 (33, 37)	38 (38, 39)	36 (34, 38)
Arterial pH	DMSO	7.45 (7.44, 7.46)	7.45 (7.39, 7.46)	7.35 (7.22, 7.41)^a^
	GW0742	7.45 (7.43, 7.46)	7.45 (7.40, 7.47)	7.36 (7.29, 7.41)^a^
Arterial base excess (mmol·L^-1^)	DMSO	1.2 (-0.3, 1.7)	1.7 (-1.1, 4.3)	-4.3 (-11.7, -0.8)^a^
	GW0742	0.4 (-0.5, 0.6)	2.5 (0.5, 4.0)	-5.3 (-7.7, -1.6)^a^
Arterial lactate (mmol·L^-1^)	DMSO	0.8 (0.7, 1.4)	1.0 (0.8, 2.0)	4.1 (1.7, 8.3)^a^
	GW0742	1.1 (0.9, 1.3)	1.0 (.7, 1.4)	2.6 (2.4, 4.5)^a^
O_2_ uptake (mL·kg^-1^·min^-1^)	DMSO	2.5 (2.2, 2.9)	3.0 (2.7, 3.1)	4.1 (3.5, 4.3)^a^
	GW0742	2.6 (2.5, 2.9)	2.7 (2.3, 3.5)	3.8 (3.4, 4.6)^a^
CO_2_ production (mL·kg^-1^·min^-1^)	DMSO	2.4 (2.2, 2.7)	2.8 (2.5, 3.0)	3.2 (3.1, 3.6)^a^
	GW0742	2.3 (2.0, 2.6)	2.6 (2.4, 3.0)	3.2 (2.8, 3.6)^a^
Blood glucose (mg·dL)	DMSO	118 (109, 127)	76 (64, 88)^a^	94 (73, 120)
	GW0742	117 (111, 122)	68 (58, 73)^a^	75 (69, 83)^a^
Glucose production (mg·kg^-1^·min^-1^]	DMSO	1.3 (1.3, 1.5)	2.1 (2.0, 2.5)	3.0 (2.4, 3.3)
	GW0742	1.3 (1.2, 1.8)	2.3 (2.1, 2.8)	3.0 (2.8, 3.5)
Glucose oxidation (mg·kg^-1^·min^-1^)	DMSO	0.4 (0.3, 0.4)	1.3 (1.2, 1.4)	1.7 (1.6, 2.1)
	GW0742	0.3 (0.3, 0.4)	1.4 (1.3, 1.6)	1.9 (1.6, 2.3)

All data are presented as median (quartiles); vehicle (DMSO): *n* = 12 (*n* = 11 during 12- to 24-h peritonitis), GW0742: *n* = 10; ^a^ *p* < 0.05 vs. before peritonitis.

**Table 2:** **Parameters of renal O**_**2**_ **exchange, metabolism, function (NGAL - neutrophil gelatinase-associated lipocalin), as well as renal venous biomarkers of inflammation (NO**_**2**_^**-**^ **+ NO**_**3**_^**-**^ **- nitrite plus nitrate) and oxidative stress (n.d. - not determined)**

		Before peritonitis	12-h peritonitis	24-h peritonitis
Renal venous PO_2_ (mmHg)	DMSO	54 (45, 61)	60 (50, 61)	56 (47, 61)
	GW0742	53 (47, 55)	58 (52, 63)	56 (48, 60)
Renal venous PCO_2_ (mmHg)	DMSO	40 (38, 41)	44 (41, 45)	45 (41, 47)
	GW0742	38 (36, 40)	44 (42, 45)	42 (37, 46)
Renal venous pH	DMSO	7.42 (7.41, 7.44)	7.42 (7.36, 7.44)	7.30 (7.12, 7.39)^a^
	GW0742	7.43 (7.40, 7.45)	7.42 (7.38, 7.44)	7.31 (7.23, 7.40)^a^
Renal venous base excess (mmol^1^·L^-1^)	DMSO	1.4 (0.0, 2.5)	2.4 (-0.2, 4.7)	-4.4 (-12.3, 1.5)^a^
	GW0742	1.0 (0.1, 1.7)	3.3 (1.3, 4.4)	-4.7 (-8.2, -2.1)^a^
Renal venous lactate (mmol^1^·L^-1^)	DMSO	0.9 (0.7, 1.4)	1.0 (0.9, 1.9)	4.1 (1.3, 9.2)^a^
	GW0742	1.0 (0.7, 1.0)	1.3 (1.2, 1.5)	3.2 (2.1, 4.3)^a^
Arterial creatinine (μmol^1^·L^-1^)	DMSO	90 (84, 96)	81 (75, 96)	126 (98, 141)^a^
	GW0742	93 (87, 95)	81 (80, 88)	124 (94, 137)^a^
Blood NGAL (ng·L^-1^)	DMSO	65 (55, 72)	n.d.	364 (270, 400)^a^
	GW0742	56 (53, 61)	n.d.	365 (276, 400)^a^
Urine output (mL·kg^-1^·h^-1^)	DMSO	8.8	(7.7, 10.4)	3.2 (1.8, 5.1)^a^
	GW0742	10.0	(8.2, 10.7)	3.6 (2.4, 6.7)^a^
Creatinine clearance (mL^1^·min^-1^)	DMSO	120	(91, 134)	56 (39, 96)^a^
	GW0742	129	(114, 140)	78 (66, 81)^a^
Fractional Na^+^ excretion (%)	DMSO	10	(8, 11)	5 (4, 8)^a^
	GW0742	8	(7, 9)	4 (3, 4)^a^
Renal venous interleukin-6 (ng·L^-1^)	DMSO	96 (77, 139)	1,710 (1,242, 4,807)^a^	8,405 (2,565, 23,153)^a^
	GW0742	97 (77, 107)	2,656 (1,186, 2,910)^a^	3,457 (3,348, 3,489)^a^
Renal venous tumor necrosis factor-α (ng·L^-1^)	DMSO	34 (28, 52)	70 (55, 105)^a^	125 (91, 172)^a^
	GW0742	49 (42, 126)	63 (50, 84)^a^	183 (131, 239)^a^
Renal venous NO_2_^-^ + NO_3_^-^ (μmol·L^-1^)	DMSO	7 (5, 14)	10 (8, 15)	14 (12, 21)^a^
	GW0742	9 (6, 10)	9 (8, 14)	17 (10, 21)^a^
Renal venous 8-isoprostane (ng·L^-1^)	DMSO	85 (80, 131)	96 (78, 132)	123 (90, 166)^a^
	GW0742	83 (72, 109)	91 (70, 113)	123 (87, 264)^a^

Data on urine output, creatinine clearance, and fractional Na^+^ excretion refer to 0 to 12 and 12 to 24 h of peritonitis, respectively. All data are presented as median (quartiles); vehicle (DMSO): *n* = 12 (*n* = 11 at 24-h peritonitis), GW0742: *n* = 10; ^a^ *p* < 0.05 vs. before peritonitis.

**Figure 1:**
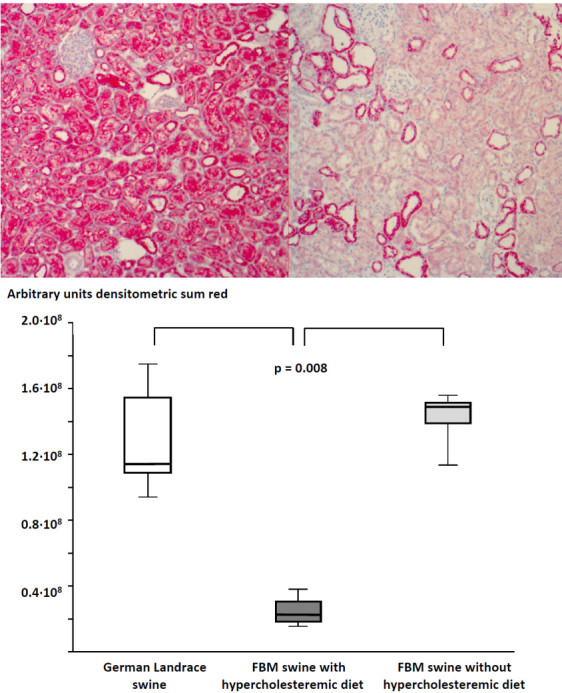
**Examples and results of the quantitative image analysis of the PPAR-β/δ immunohistochemistry in biopsies.** Examples **(A)** (magnification ×20; left panel: German Landswine, right panel: FBM swine) and results of the quantitative image analysis **(B)** of the PPAR-β/δ immunohistochemistry in biopsies taken during surgical instrumentation in comparison to biopsies taken in otherwise young and healthy German Landswine undergoing a similar surgical instrumentation. German Landswine (open box-and-whisker plots), FBM swine with (dark gray box-and-whisker plots), and without hyperchloesteremic diet (light gray box-and-whisker plots). All data are median (quartiles, range; *n* = 5 in each group).

Kidney histopathology showed only mild to moderate glomerular and tubular damage without inter-group difference (Additional file 1: Table C). Immunohistochemistry showed marked nitrotyrosine formation, again without inter-group difference (Additional file 1: Table B). Immunohistochemistry of the renal PPAR-β/δ showed that expression was nearly ten times lower than both in young and healthy German Landswine and age-matched FBM pigs that had not been fed with the atherogenic diet (Figure 1).

## Discussion

This study was to test the hypothesis whether the PPAR-β/δ agonist GW0742 would attenuate kidney injury during long-term, resuscitated, polymicrobial porcine septic shock. Since GW0742 had been effective in animals with obesity and diabetes, we studied swine with hyperlipidemia and ubiquitous atherosclerosis [15]. The major findings were that (1) GW0742 failed to attenuate sepsis-induced organ dysfunction and histological damage and (2) did not affect the parameters of inflammation and oxidative and nitrosative stress.

The lacking efficacy of GW0742 is in contrast to previous studies in polymicrobial sepsis [2, 7], which, however, report data from young and otherwise healthy rodents. We studied septic shock in FBM swine, a strain presenting with ubiquitous hypercholesteremia-induced atherosclerosis [15]. In addition, these animals showed a several-fold reduction of the kidney PPAR-β/δ expression as compared to young and healthy German Landswine. In obese, insulin-resistant mice, weight loss increased PPAR-γ expression [17], and both starvation and endurance training activated PPAR-δ in healthy animals [18, 19]. PPAR-δ knockout mice are glucose-intolerant [11], and PPAR-β/δ activation normalized the diabetes-related endothelial dysfunction [12]. Our FBM swine presented with both hypercholesteremia as well as impaired glucose homoeostasis; at baseline, direct, aerobic glucose oxidation was significantly lower (<30% vs. 60% to 75% of glucose production) when compared to young (age 3 to 4 months) and healthy German Landswine undergoing the same protocol of resuscitated fecal peritonitis [13, 14]. Hence, the pre-existing atherosclerosis together with the 'metabolic syndrome’ may have caused GW0742 inefficacy due to PPAR-β/δ down-regulation. It could be argued in this context that GW0742 did attenuate ischemia/reperfusion injury in obese and diabetic rats [9, 10]. These data, however, originate from un-resuscitated, short-term models in rats, whereas we studied long-term, fully resuscitated porcine septic shock.

Our findings of reduced PPAR-β/δ expression were obtained from biopsies taken during surgical instrumentation, i.e., prior to any inflammatory challenge [15]. Since the abdominal cavity had been closed again after the surgical instrumentation, we could not obtain kidney biopsies after induction of peritonitis. Therefore, we cannot exclude that sepsis further aggravated PPAR-β/δ down-regulation and thus contributed to the lacking efficacy of GW0742. In rodents, any shock-related effect on PPAR expression seems to be organ-specific; hemorrhage, injection of endotoxin, or cecal ligation and puncture significantly reduced pulmonary [20], myocardial [21], and hepatic [22] PPAR-α, -γ, or -δ protein expression and/or mRNA. However, endotoxemia only decreased renal PPAR-α mRNA, whereas PPAR-γ and PPAR-δ mRNA remained unchanged [23].

### Limitations of the study

Since we did not study the effect of GW0742 in young and healthy animals, we do not have direct evidence that the PPAR-β/δ down-regulation associated with the underlying hypercholesteremia and atherosclerosis rather than other potentially confounding factors contributed to our findings, e.g., the use of adult swine *per se*, the integration of standard intensive care procedures into the experimental design, the timing and dosing of the treatment, and/or the duration of the study. To our knowledge, GW0742 has only been studied in rodents. Nevertheless, GW501516, another highly specific PPAR-β/δ agonist with a chemical structure very close to that of GW0742 [1], attenuated endotoxin-induced NF-κB activation, and cytokine release not only in rodent [24–26] but also in human tissues [26, 27]. So far, a modulation of the PPAR-β/δ expression related to nutritional and/or metabolic interventions has only been studied in rodents as well [18, 19]. However, in swine, a similar diet-induced hypercholesteremia as in our experiments [28] was associated with a markedly reduced PPAR-α and PPAR-γ expression [29], coinciding with impaired glucose tolerance [28, 29], arterial hypertension [28], increased transaminase activities [29], and aggravated oxidative stress [28]. Treatment with resveratrol restored PPAR-α and PPAR-γ expression [29], going along with improved left heart perfusion and function during chronic myocardial ischemia [30, 31]. It could be argued that all rodent data on GW0742 were obtained in young animals, while we studied adult swine. In fact, ischemia/reperfusion injury and hemorrhagic shock induced PPAR-γ expression only in young but not in adult or old rodents [32, 33], which in turn impaired adaptive autophagy [33]. There are no data available on PPAR-β/δ expression under these conditions, but the above-mentioned studies on hypercholesteremia-induced PPAR-α and PPAR-γ down-regulation also originate from adult swine. Moreover, tissue PPAR-β/δ expression in kidneys from adult FBM swine that had not been fed with cholesterol-enriched diet was similar to that in young and healthy German Landswine (Figure 1B). We only studied the effect of 0.03 mg∙kg^-1^ of GW0742. This dose was chosen because it attenuated kidney dysfunction in murine endotoxin- and cecal ligation and puncture-induced septic shock at plasma concentrations selectively activating the PPAR-β/δ without any cross-reactivity on the other PPAR isoforms [2]. Moreover, in rats, this dose reduced organ injury after kidney ischemia/reperfusion injury [10], and a ten times higher dose did not further influence myocardial ischemia/reperfusion injury [3]. It should be noted in this context that equipotent drug doses are usually much lower on a per kilogram basis in large species (e.g., swine, dogs) than in rodents [34]. FBM swine present with reduced creatinine clearance and moderate histological damage of the kidney already under baseline conditions [15]. Nevertheless, any pre-existing organ dysfunction most likely did not influence our results; due to fluid resuscitation and catecholamine infusion, creatinine clearance was comparable to that in young and healthy German Landswine undergoing the same protocol of resuscitated fecal peritonitis [13, 14].

## Conclusions

In swine with pre-existing atherosclerosis, the PPAR-β/δ agonist GW0742 failed to attenuate septic shock-induced kidney dysfunction and organ damage, most likely due to reduced receptor expression associated with cardiovascular and metabolic co-morbidity.

## Electronic supplementary material

Additional file 1: **Supplementary information.** Table A: Norepinephrine infusion rate, hydroxyethyl starch infusion rate and urine output. Table B: Activation of NF-κB, protein expression of the iNOS, HO-1, cleaved caspase-3, and nitrotyrosine in *post mortem* kidney specimen. Table C: Kidney histopathology score. Figure A: Representative examples of the histopathological items analyzed. (DOCX 1 MB)
