# Supplementary material for: Effects of the PPAR-β/δ agonist GW0742 during resuscitated porcine septic shock
Source: Intensive Care Med Exp. 2013 Oct 29;1:9. doi: 10.1186/2197-425X-1-9 (PMC4796150; doi:10.1186/2197-425X-1-9)

Additional file 1

**Effects of the PPAR-**β**/**δ **agonist GW0742 during resuscitated porcine septic shock**

Martin Wepler, Sebastian Hafner, Matthias Reize, Angelika Scheuerle, Oscar McCook, Ulrich Wachter, Josef A. Vogt, Michael Gröger, Florian Wagner, Florian Simon, José Matallo, Frank Gottschalch, Andrea Seifritz, Bettina Stahl, Martin Matejovic, Amar Kapoor, Peter Möller, Enrico Calzia, Michael Georgieff, Peter Radermacher, Christoph Thiemermann

Additional online data:

Table A: Norepinephrine infusion rate, hydroxyethyl starch infusion rate and urine output.

Table B: Activation of NF-κB, protein expression of the iNOS, HO-1, cleaved caspase-3, and nitrotyrosine in *post mortem* kidney specimen.

Table C: Kidney histopathology score.

Figure A: Representative examples of the histopathological items analyzed.

**Methods:**

*Immune histochemistry of the PPAR-*β*/*δ

The PPAR-β/δ expression was determined on formalin-fixed, paraffin-embedded kidney biopsies, which had been taken during surgical instrumentation both in FBM and young and healthy German Landswine in a previous study [15], as well as aged matched FBM pigs that had not been fed with the atherogenic diet (n = 5). R. These biopsies were taken from the cortex of the un-instrumented kidney and immediately placed in fixative. Post-mortem biopsies were taken immediately at the time of death. The kidney capsule was removed and the kidney sectioned sagitally. Three 20 mm x 4 mm pyramid shape samples from the middle region of the kidney including cortex, medulla, renal papilla and the renal calyx were dissected, placed in a minimum of 10 times the volume of formalin (4% Buffered Paraformaldehyde) in tissue tek embedding cassets, and fixed for six days before dehydrating and embedding in paraffin. Sections (3 μm) were cut from each sample and deparaffinised in xylene and graded ethanol to water as follows: four times incubation in xylene, twice in 100% ethanol for 5 minutes, once in 90 %, 70 %, and 40 % ethanol, each, for five minutes, and another five minutes in distilled 100% H2O.

*Heat-induced epitope retrieval (HIER)*

10mM citrate puffer pH 6.0 (2X) 5min microwaving after cooling slides were rinsed for 2-3 minutes dH20. Slides were then blocked for 30 minutes in 10% normal goat sera in TBS. The primary antibody was a rabbit polyclonal PPAR delta 1:100 (#ab23673, Abcam plc, Cambridge, UK) diluted in TBS pH 8.0 with .001 % Tween 20 incubated at room temperature for one hour. Detection was performed using the Dako REAL™ Detection System, Alkaline Phosphatase/Red, rabbit Dako Corp. following manufacturers recommendation. Briefly, Dako A = biotinylated secondary goat anti-rabbit antibody incubation at room temperature for 30 minutes; Dako B = strepavidin-alkaline phosphatase incubation at room temperature for 30 minutes; Dako F = red chromogen (alkaline phosphatase substrate) incubated at room temperature for 15 minutes. Counterstaining was done for one minute with Mayers hematoxylin.

*Image analysis*

Slides were visualized using a Zeiss Axio Imager A1 microscope, using a 10x objective (EC Plan-NEOFLUAR). Three distinct representative cortical (800,000 μm²) regions were quantified densitometrically for intensity immunoreactive regions using the AxioVision (rel. 4.8) software (Zeiss, Jena, Germany). Results are presented as mean densitometric sum red.

**Table A:** Norepinephrine infusion rate (mean infusion rate during the whole experiment) as well as hydroxyethyl starch infusion rate and urine output during the two phases of septic shock. All data median (quartiles), vehicle (DMSO): n = 12 (n = 11 during 12 – 24 hours peritonitis), GW0742: n = 10, # designates p < 0.05 vs. 0 – 12 hours peritonitis.

|  |  | 0 – 12 hours peritonitis | 12 – 24 hours peritonitis |
| --- | --- | --- | --- |
| Norepinephrine infusion rate  [μg·kg^-1^·min^-1^] | DMSO  GW0742 | 0.92 (0.36;2.67)  0.56 (0.46;0.91) | |
| Hydroxyethyl starch infusion rate  [mL·kg^-1^·h^-1^] | DMSO  GW0742 | 10 (9;10)  10 (10;10) | 8 (8;10)  10 (9;10) |
| Urine output  [mL·kg^-1^·h^-1^] | DMSO  GW0742 | 8.8 (7.7;10.4)  10.0 (8.2;10.7) | 3.2 (1.8;5.1) #  3.6 (2.4;6.7) # |

**Table B.** Activation of the nuclear transcription factor κB (NF-κB), protein expression of the inducible nitric oxide synthase (iNOS), heme oxygenase-1 (HO-1), and cleaved caspase-3 (as fold increase of control values from animals which had only undergone surgical instrumentation), and nitrotyrosine (arbitrary units of densitometric red staining) in immediate *post mortem* kidney biopsies. All data median (quartiles), vehicle (DMSO): n = 12, GW0742: n = 10.

| NF-κB | DMSO  GW0742 | 2.6 (2.2;2.7)  2.6 (2.3;3.0) |
| --- | --- | --- |
| iNOS | DMSO  GW0742 | 0.9 (0.9;1.1)  1.0 (0.9;1.1) |
| HO-1 | DMSO  GW0742 | 1.0 (1.0;1.1)  1.0 (0.9;1.0) |
| Cleaved  caspase-3 | DMSO  GW0742 | 1.1 (1.0;1.2)  1.1 (1.0;1.1) |
| Nitrotyrosine  [arbitrary units] | DMSO  GW0742 | 4.8 (3.4;6.1)·10^8^  6.2 (0.1;7.8)·10^8^ |

**Table C.** Kidney histopathology score. Glomerular tubularization, i.e. the herniation of proximal tubular epithelial cells into Bowman’s capsule along the luminal surface of the capsule, is reported as the number of glomeruli showing herniation of the tubular epithelium in % of all glomeruli analyzed; all other data are the mean value of the scores of the five random sections for each item analyzed. All data median (quartiles), vehicle (DMSO): n = 12, GW0742: n = 10.

|  | DMSO | GW0742 |
| --- | --- | --- |
| % Glomerular tubularization | 7 (2;11) | 4 (1;7) |
| Tubular apoptosis/necrosis | ∅ | ∅ |
| Protein cylinders | 0 (0;1) | 0 (0;0) |
| Dilatation of Bowman’s space | 2 (1;2) | 1 (0;2) |

**Figure A:** Typical examples (HE staining, magnification 20x) of “*glomerular tubularization*”, i.e. the herniation of proximal tubular epithelial cells into Bowman’s capsule along the luminal surface of the capsule (a), dilatation of Bowman’s space (b), and swelling of Bowman’s capsule (c), cellular edema of the proximal tubule (d), distal tubular dilatation and elongation (e), and fibrin deposit (f). Arrows indicate the specific histo-pathological item.


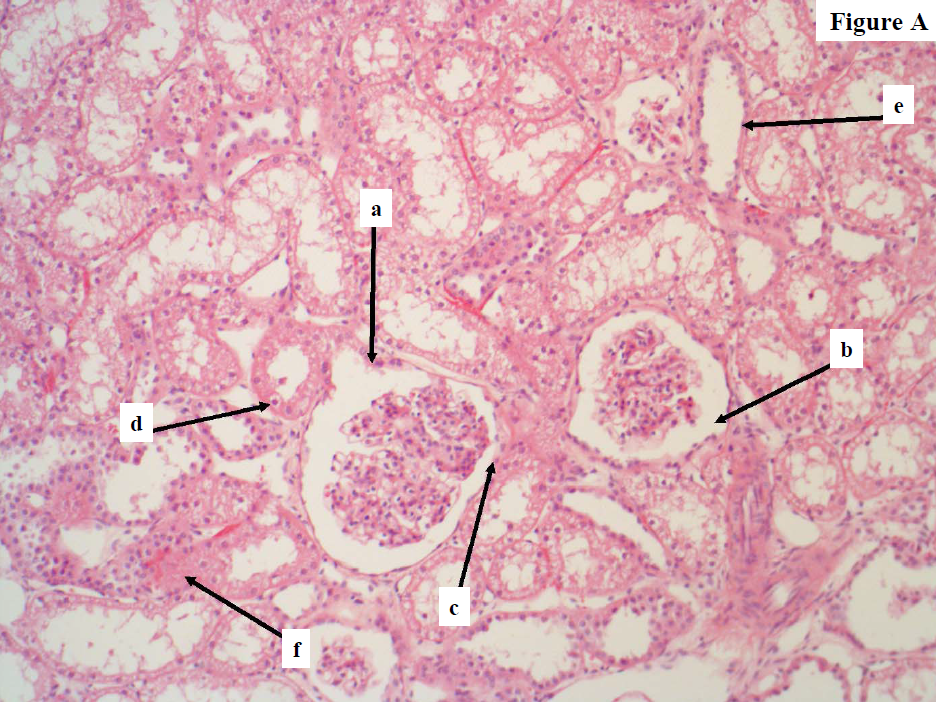

Supplement: Supplementary file 1 — Additional file 1: Supplementary information. Table A: Norepinephrine infusion rate, hydroxyethyl starch infusion rate and urine output. Table B: Activation of NF-κB, protein expression of the iNOS, HO-1, cleaved caspase-3, and nitrotyrosine in post mortem kidney specimen. Table C: Kidney histopathology score. Figure A: Representative examples of the histopathological items analyzed. (DOCX 1 MB) [file 40635_2013_28_MOESM1_ESM.docx]
